# A study of cortical and brainstem mechanisms of diffuse noxious inhibitory controls in anaesthetised normal and neuropathic rats

**DOI:** 10.1111/ejn.14576

**Published:** 2019-10-06

**Authors:** Ryan Patel, Anthony H. Dickenson

**Affiliations:** ^1^ Department of Neuroscience, Physiology and Pharmacology University College London London UK

**Keywords:** descending pain modulation, dorsal horn, dorsal reticular nucleus, in vivo electrophysiology, infralimbic cortex, spinal nerve ligation

## Abstract

Diffuse noxious inhibitory controls (DNIC) are a mechanism of endogenous descending pain modulation and are deficient in a large proportion of chronic pain patients. However, the pathways involved remain only partially determined with several cortical and brainstem structures implicated. This study examined the role of the dorsal reticular nucleus (DRt) and infralimbic (ILC) region of the medial prefrontal cortex in DNIC. In vivo electrophysiology was performed to record from dorsal horn lamina V/VI wide dynamic range neurones with left hind paw receptive fields in anaesthetised sham‐operated and L5/L6 spinal nerve‐ligated (SNL) rats. Evoked neuronal responses were quantified in the presence and absence of a conditioning stimulus (left ear clamp). In sham rats, DNIC were reproducibly recruited by a heterotopically applied conditioning stimulus, an effect that was absent in neuropathic rats. Intra‐DRt naloxone had no effect on spinal neuronal responses to dynamic brush, punctate mechanical, evaporative cooling and heat stimuli in sham and SNL rats. In addition, intra‐DRt naloxone blocked DNIC in sham rats, but had no effect in SNL rats. Intra‐ILC lidocaine had no effect on spinal neuronal responses to dynamic brush, punctate mechanical, evaporative cooling and heat stimuli in sham and SNL rats. However, differential effects were observed in relation to the expression of DNIC; intra‐ILC lidocaine blocked activation of DNIC in sham rats but restored DNIC in SNL rats. These data suggest that the ILC is not directly involved in mediating DNIC but can modulate its activation and that DRt involvement in DNIC requires opioidergic signalling.

## Graphical abstract

Neuropathic rats exhibit deficits in endogenous pain modulation as shown by a loss of diffuse noxious inhibitory controls (DNIC). In sham rats, the expression of DNIC requires opioidergic signalling in the dorsal reticular nucleus. In addition, the infralimbic cortex exerts bidirectional control of DNIC depending on pain state revealing novel pathophysiological mechanisms.

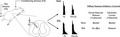

**Abbreviations**
CI: confidence interval
CPM: conditioned pain modulation
DNIC: diffuse noxious inhibitory controls
DRt: dorsal reticular nucleus
ILC: infralimbic cortex
RM: repeated measures
SNL: spinal nerve ligated
WDR: wide dynamic range

## 1 INTRODUCTION

The adage that “pain inhibits pain” is underpinned by diffuse noxious inhibitory controls (DNIC)—a descending pain modulatory mechanism, recruited by a distant noxious stimulus, that can suppress firing of convergent second‐order sensory neurones (Le Bars, Dickenson, & Besson, 1979). The human counterpart measure, now referred to as conditioned pain modulation (CPM), is considered to be the psychophysical outcome of activating DNIC, and has received renewed interest in recent years as a sensory testing tool. CPM/DNIC likely reflect the net balance between descending inhibitory and facilitatory signalling; hence, the study of DNIC in rodents represents a useful translatable measure linking pre‐clinical and clinical investigations (Bannister & Dickenson, 2017). Inefficient CPM might provide insight into underlying pathophysiological mechanisms, and disturbances have been reported in neuropathic pain, irritable bowel syndrome, cluster headache and fibromyalgia (Albusoda et al., 2018; Kosek & Hansson, 1997; Perrotta et al., 2013; Yarnitsky, Granot, & Granovsky, 2014). This proposal has also garnered support of mechanism‐led treatment of neuropathic patients as CPM efficiency inversely correlates with pain relief from tapentadol and duloxetine (Niesters et al., 2014; Yarnitsky, Granot, Nahman‐Averbuch, Khamaisi, & Granovsky, 2012). Drawing parallels with rodent studies, tapentadol restores absent DNIC in neuropathic rats (Bannister, Patel, Goncalves, Townson, & Dickenson, 2015), but fails to enhance functional DNIC in uninjured rats (Lockwood & Dickenson, 2019). Furthermore, pre‐operative patients with low CPM levels were at greater risk of developing chronic post‐operative pain (Wilder‐Smith, Schreyer, Scheffer, & Arendt‐Nielsen, 2010; Yarnitsky et al., 2008), consistent with animal data demonstrating that susceptibility to pain chronicity after nerve injury related to the ability to engage descending inhibitory pathways (De Felice et al., 2011; Xu, Kontinen, & Kalso, 1999).

The neural networks that subserve DNIC appear partially distinct to the more established and characterised descending pain modulatory network. In terms of ascending pathways, activation of parabrachial‐projecting NK1 + spinal neurones is required to recruit both pontospinal and bulbospinal modulatory pathways (Rahman, Suzuki, Hunt, & Dickenson, 2008; Suzuki, Morcuende, Webber, Hunt, & Dickenson, 2002), whereas both spinoparabrachial and spinoreticular pathways are involved in activating DNIC (Lapirot et al., 2009; Suzuki et al., 2002; Villanueva, Peschanski, Calvino, & Le Bars, 1986). In the descending arm of the loop, perhaps surprisingly, DNIC are conserved following lesioning of the periaqueductal grey, rostral ventromedial medulla and locus coeruleus (Bouhassira, Bing, & Le Bars, 1990, 1992; Bouhassira, Chitour, Villanueva, & Le Bars, 1993), but are diminished following lesion of the dorsal reticular nucleus (DRt), also referred to as the subnucleus reticularis dorsalis (Bouhassira, Villanueva, Bing, & le Bars, 1992). However, more recent studies confirm noradrenergic signalling comprises a significant component of DNIC (Bannister et al., 2015; Peters et al., 2015; Wen et al., 2010), but also implicate a broader role of descending monoaminergic signalling systems (Chebbi et al., 2014; Lapirot et al., 2011).

Imaging studies in pain‐free individuals reveal that cortical influences on brainstem circuitry determine conditioned pain modulation (Piche, Arsenault, & Rainville, 2009; Sprenger, Bingel, & Buchel, 2011; Youssef, Macefield, & Henderson, 2016a,b) and that low CPM was associated with enhanced functional connectivity between the prefrontal cortex and DRt (Youssef et al., 2016a). Rodent studies of cortical involvement in DNIC are lacking, and the precise mechanisms within the DRt are not fully understood. DNIC is partly mediated via an opioidergic mechanism (Le Bars, Chitour, Kraus, Dickenson, & Besson, 1981), and opioidergic interneurones in the DRt receive projections from multiple cortical regions (Martins et al., 2015a). These interneurones might be recruited during DNIC, and we investigated this possibility by inhibiting with naloxone. By silencing with lidocaine, we additionally investigated whether the infralimbic (ILC) region of the medial prefrontal cortex (mPFC) forms part of DNIC circuitry in rats with functional DNIC, and in a model of neuropathy characterised by an absence of DNIC (Bannister, Lockwood, Goncalves, Patel, & Dickenson, 2017; Bannister et al., 2015).

## 2 MATERIALS AND METHODS

### 2.1 Animals

Sham or spinal nerve‐ligated (14–18 days post‐surgery) male Sprague‐Dawley rats (250–300 g) were used for electrophysiological experiments (Biological Services, University College London, UK). Animals were group‐housed (maximum of 4) on a conventional 12‐hr: 12‐hr light–dark cycle; food and water were available ad libitum. Temperature (20–22°C) and humidity (55%–65%) of holding rooms were closely regulated. All procedures described here were approved by an internal ethics committee and sanctioned by the UK Home Office (licence IEEC97183), adhered to the Animals (Scientific Procedures) Act 1986/directive 2010/63/EU, and were designed in accordance with ethics guidelines outlined by the International Association for the Study of Pain (Zimmermann, 1983). A total of 12 sham and 13 SNL rats were used in this study; one neurone was recorded per rat.

### 2.2 Spinal nerve ligation (SNL) surgery

Spinal nerve ligation surgery was performed as previously described (Ho Kim & Mo Chung, 1992). Rats (130–140 g) were maintained under 2% v/v isoflurane anaesthesia delivered in a 3:2 ratio of nitrous oxide and oxygen. Under aseptic conditions, a paraspinal incision was made and the tail muscle retracted from the spinal column. Part of the L5 transverse process was removed to expose the left L5 and L6 spinal nerves, which were then isolated with a glass nerve hook (Ski‐Ry, London, UK) and ligated with a non‐absorbable 6‐0 braided silk thread proximal to the formation of the sciatic nerve. The surrounding skin and muscle was closed with absorbable 4‐0 sutures, and lidocaine cream (5% w/w) was applied topically to the closed incision. Sham surgery was performed in an identical manner omitting the nerve isolation and ligation step. All rats groomed normally and gained weight in the following days post‐surgery.

### 2.3 In vivo electrophysiology

**Figure 1:**
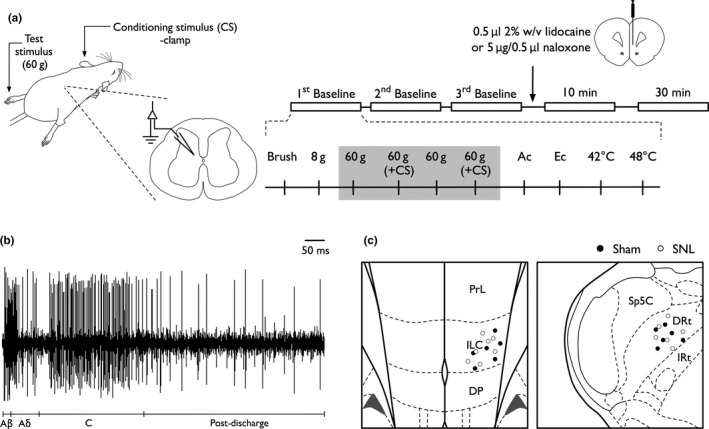
Experimental protocol for single‐unit dorsal horn recordings (a). A range of low intensity and noxious mechanical and thermal stimuli were applied to the receptive field approximately 50–60 s apart. The effect of a concurrently applied conditioning stimulus (CS; noxious clamp applied to left ear) was tested on the neuronal response to a test stimulus (60 g von Frey); individual baseline values represent mean of two tests (grey box). Following drug delivery, stimulus‐evoked responses were quantified at 10 and 30 min post‐dosing. Spike trace of a deep dorsal horn wide dynamic range neurone following electrical stimulation of the receptive field at 3 times the C‐fibre threshold; note afferent‐evoked activity in the Aβ, Aδ and C‐fibre conduction range (b). Schematic representation of tract termination sites (bregma +3 and −13.9 images chosen for illustrative purposes) (c). Ac, acetone; Ec, ethyl chloride; DRt, dorsal reticular nucleus; ILC, infralimbic cortex

Anaesthesia was initially induced with 3.5% v/v isoflurane delivered in 3:2 ratio of nitrous oxide and oxygen. Once areflexic, a tracheotomy was performed and rats were subsequently maintained on 1.5% v/v isoflurane for the remainder of the experiment (approximately 3–4 hr; core body temperature was maintained throughout with the use of a homeothermic blanket). Rats were then secured in a stereotaxic frame, a midline incision was made across the scalp, and after the skull‐exposed co‐ordinates for either the ILC or DRt were calculated in relation to bregma (Watson & Paxinos, 2006). A small craniotomy was performed with a high‐speed surgical micro‐drill. A laminectomy was subsequently performed to expose the L4–L6 segments of the spinal cord, and two spinal clamps were applied to stabilise the spinal column. Extracellular recordings were obtained from deep dorsal horn wide dynamic range (WDR) lamina V/VI neurones with receptive fields on the glabrous skin of the left hind toes using 127‐μm‐diameter 2 MΩ parylene‐coated tungsten electrodes (A‐M Systems, Sequim, WA). The search stimulus consisted of light tapping of the left hind paw as the electrode was manually lowered. Neurones were characterised from depths relating to deep dorsal horn laminae (sham: 752 ± 94 μm; SNL: 626 ± 49 μm) (Watson, Paxinos, Kayalioglu, & Heise, 2009) and were classified as WDR on the basis of sensitivity to dynamic brushing, and noxious mechanical (60 g) and heat stimulation (48°C) of the receptive field. WDR neurones recorded at these depths receive convergent Aβ, Aδ and C‐fibre input (Figure 1b), as previously observed (Patel, Kucharczyk, Montagut‐Bordas, Lockwood, & Dickenson, 2019). The signal was amplified (×3,000) and bandpass‐filtered (low/high‐frequency cut‐off 150/2,000 Hz); data were captured and analysed by a CED1401 interface coupled to a computer with Spike2 v4 software (Cambridge Electronic Design, Cambridge, United Kingdom).

Figure 1a summarises the experimental protocol. The receptive field was stimulated using a range of natural stimuli (brush, von Frey filaments 8 and 60 g, and heat 42, and 48°C) applied over a period of 10 s per stimulus. The heat stimulus was applied with a constant water jet onto the centre of the receptive field. Acetone and ethyl chloride (100 μl) were applied as an evaporative innocuous cooling and noxious cooling stimulus, respectively (Leith, Koutsikou, Lumb, & Apps, 2010), and responses quantified over 10 s post‐application. Evoked responses to room temperature water (25°C) were minimal, or frequently completely absent, and subtracted from acetone and ethyl chloride evoked responses to control for any concomitant mechanical stimulation during application. A noxious clamp (using a 35‐mm bulldog serrefine (InterFocus, Linton, UK)) was applied to the left ear as a conditioning stimulus concurrently to stimulation of the hind paw with a 60 g von Frey filament. In this and previous studies (Bannister et al., 2015; Bannister, Lockwood, et al., 2017), we have set the conditioning stimulus at a level to produce sub‐maximal DNIC in order to align the effect size with CPM in humans (Nir, Granovsky, Yarnitsky, Sprecher, & Granot, 2011). After three consecutive stable baseline responses to evoked stimuli (data were averaged to give control values), 0.5 μl 2% w/v lidocaine (Sigma, Gillingham, UK) or 5 μg/0.5 μl naloxone hydrochloride (Sigma, Gillingham, UK) dissolved in normal saline was injected into the ILC (RC + 3 mm, ML −0.6 mm, DV −5.2 mm) and DRt (RC −13.9 mm, ML + 1.7 mm, DV −8.4 mm), respectively. Correct placement of the drug was verified after sectioning of brains (Figure 1c). Neuronal responses to mechanical and thermal stimuli were tested at 10 and 30 min post‐dosing; for all data sets, the 10 min of time point is plotted. The injection volume and time points were chosen to mitigate the effect of drug diffusion (likely to be between 0.5 and 1 mm); however, drug effects in neighbouring brain regions cannot be ruled out. All drug effects were transient indicating that these cannot be attributed to tissue damage caused by drug delivery. Injection of 0.5 μl saline into the neighbouring anterior cingulate cortex (Bannister, Qu, et al., 2017) or rostral ventromedial medulla (unpublished observation) does not affect spinal neuronal excitability, in contrast to gabapentin or lidocaine injection, respectively, supporting that the injection volume alone does not disrupt normal cortical and brainstem function.

### 2.4 Statistics

Statistical analyses were performed using SPSS v25 (IBM, Armonk, NY). Drug effects on DNIC, and heat and mechanical coding of neurones were compared with a 2‐way repeated‐measures (RM) ANOVA, followed by a Bonferroni *post hoc* test for paired comparisons. Where appropriate, sphericity was tested using Mauchly's test; the Greenhouse–Geisser correction was applied if violated. Collated baseline DNIC responses, and cold‐ and brush‐evoked firing were compared with two‐tailed paired Student's *t* test. All data represent mean ± 95% confidence interval (CI). * *p *< .05, **p < .01, ***p < .001.

## 3 RESULTS

### 3.1 DNIC are abolished by naloxone injection into the dorsal reticular nucleus in sham rats but are unaltered in neuropathic rats

**Figure 2:**
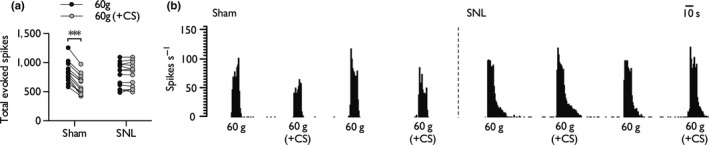
DNIC are active in sham‐operated rats but deficient in a neuropathic state. Collated baseline single‐unit neuronal responses to 60 g von Frey stimulation in the absence of and presence of a heterotopic conditioning stimulus (CS) in sham and SNL rats (a). Histogram traces depict representative neuronal responses during a baseline trial (b). Sham: *n *= 12, SNL *n *= 13. Asterisks (*) denote difference from control response, ***p < .001

Heterotopic application of a noxious ear clamp reliably and reproducibly activated DNIC in sham rats as demonstrated by a reduction in neuronal firing (60 g: 842.6 ± 117.4 spikes; 60 g + CS: 631.1 ± 109 spikes, 25.1% decrease, Cohen's *d *= −1.186) in response to a test stimulus (*t *= 13.583, *df *= 11, *p *= .00000003; Figure 2a, b). In contrast, the presence of a conditioning stimulus had no effect on neuronal responses (60 g: 797.4 ± 125.8 spikes; 60 g + CS: 797 ± 126.2 spikes, 0.005% increase, Cohen's *d *= 0.018) to the test stimulus in SNL rats (*t *= 0.043, *df *= 12, *p *= .966) (Figure 2a, b).

**Figure 3:**
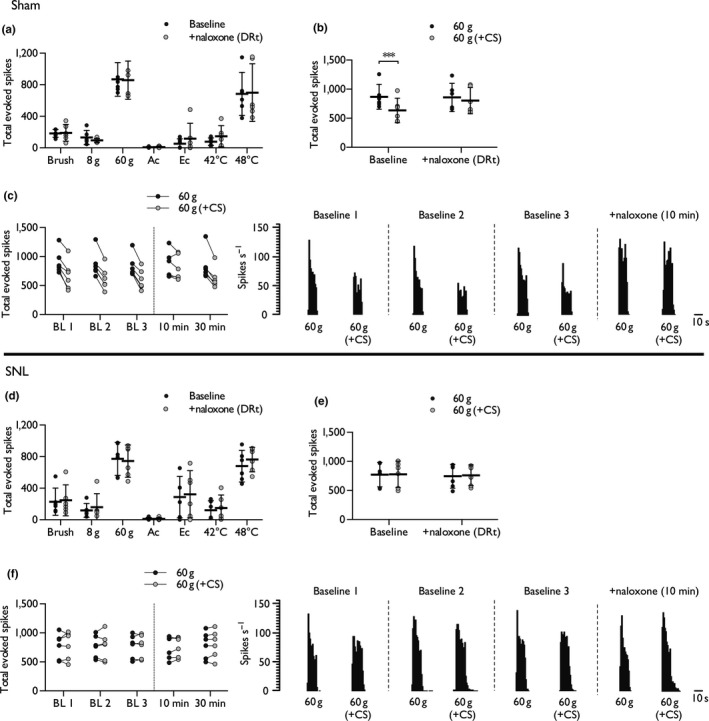
Intra‐DRt naloxone blocks the expression of DNIC in sham rats but has no effect in SNL rats. Effect of intra‐DRt naloxone on mechanical‐, cold‐ and heat‐evoked spinal neuronal responses, in the absence of conditioning, in sham rats (a). Effect of intra‐DRt naloxone on the expression of DNIC in sham rats (b), and time course of corresponding single‐unit responses pre‐ and post‐dosing (c). Effect of intra‐DRt naloxone on mechanical‐, cold‐ and heat‐evoked spinal neuronal responses, in the absence of conditioning, in SNL rats (d). Effect of intra‐DRt naloxone on the expression of DNIC in SNL rats (e), and time course of corresponding single‐unit responses pre‐ and post‐dosing (f). Histogram traces represent typical single‐unit responses. Sham: *n *= 6, SNL *n *= 6; data represent mean ± 95% CI. Asterisks (*) denote difference from control response, ***p < .001. Ac, acetone; BL, baseline; CS, conditioning stimulus; DRt, dorsal reticular nucleus; Ec, ethyl chloride

The impact of intra‐DRt naloxone injection on spinal neuronal excitability (in the absence of conditioning) was examined. Blocking opioidergic signalling in the DRt of sham rats had no effect on spinal neuronal responses to dynamic brushing (*t *= −0.222, *df *= 5, *p *= .833), punctate mechanical (2‐way RM ANOVA, main effect: *F*_1,5_ = 0.237, *p *= .939), innocuous (acetone: *t *= −1.715, *df *= 5, *p *= .147) and noxious (ethyl chloride: *t *= −1.129, *df *= 5, *p *= .647) evaporative cooling, and heat stimulation (2‐way RM ANOVA, main effect: *F*_1,5_ = 0.371, *p *= .569; Figure 3a). However, the expression of DNIC at the spinal level was abolished by intra‐DRt naloxone (2‐way RM ANOVA, interaction: *F*_1,5_ = 22.89, *p *= .005; Figure 3b, c). Likewise, in SNL rats intra‐DRt naloxone injection had no effect on spinal neuronal responses to dynamic brush (*t *= −1.499, *df *= 5, *p *= .194), punctate mechanical (2‐way RM ANOVA, main effect: *F*_1,5_ = 0.033, *p *= .862), innocuous (acetone: *t *=* *0.461, *df *=* *5, *p *=* *.664) and noxious (ethyl chloride: *t *=* *−1.942, *df *=* *5, *p *=* *.11) evaporative cooling, and heat stimulation (2‐way RM ANOVA, main effect: *F*_1,5_ = 3.941, *p *=* *.104; Figure 3d). In addition, intra‐DRt naloxone did not alter the expression of DNIC in SNL rats (2‐way RM ANOVA, interaction: *F*_1,5_ = 0.127, *p *=* *.736; Figure 3e, f).

### 3.2 DNIC are abolished by lidocaine injection into the infralimbic cortex in sham rats but restored in neuropathic rats

**Figure 4:**
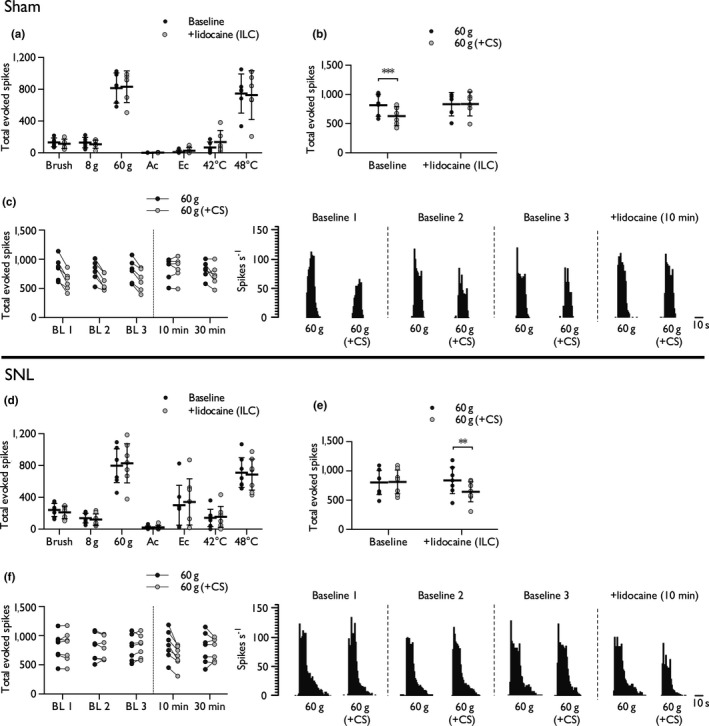
Intra‐ILC lidocaine blocks the expression of DNIC in sham rats but restores DNIC in SNL rats. Effect of intra‐ILC lidocaine on mechanical‐, cold‐ and heat‐evoked spinal neuronal responses, in the absence of conditioning, in sham rats (a). Effect of intra‐ILC lidocaine on the expression of DNIC in sham rats (b), and time course of corresponding single‐unit responses pre‐ and post‐dosing (c). Effect of intra‐ILC lidocaine on mechanical‐, cold‐ and heat‐evoked spinal neuronal responses, in the absence of conditioning, in SNL rats (d). Effect of intra‐ILC lidocaine on the expression of DNIC in SNL rats (e), and time course of corresponding single‐unit responses pre‐ and post‐dosing (f). Histogram traces represent typical single‐unit responses. Sham: *n *= 6, SNL *n *= 7; data represent mean ± 95% CI. Asterisks (*) denote difference from control response, **p < .01, ***p < .001. Ac, acetone; BL, baseline; CS, conditioning stimulus; Ec, ethyl chloride; ILC, infralimbic cortex

The impact of intra‐ILC lidocaine injection on spinal neuronal excitability (in the absence of conditioning) was examined. Blocking activity in the ILC of sham rats had no effect on spinal neuronal responses to dynamic brushing (*t *=* *0.76, *df *=* *5, *p *=* *.482), punctate mechanical (2‐way RM ANOVA, main effect: *F*_1,5_ = 0.007, *p* = .939), innocuous (acetone: *t *=* *−0.865, *df *=* *5, *p *=* *.427) and noxious (ethyl chloride: *t *=* *−1.386, *df *=* *5, *p *=* *.224) evaporative cooling, and heat stimulation (2‐way RM ANOVA, main effect: *F*_1,5_ = 0.356, *p *=* *.577; Figure 4a). The expression of DNIC at the spinal level was abolished by intra‐ILC lidocaine (2‐way RM ANOVA, interaction: *F*_1,5_ = 69.09, *p *=* *.00041; Figure 4b, c). Likewise, in SNL rats intra‐ILC lidocaine injection had no effect on spinal neuronal responses to dynamic brush (*t *=* *1.715, *df *=* *6, *p* = .137), punctate mechanical (2‐way RM ANOVA, main effect: *F*_1,6_ = 0.079, *p* = .788), innocuous (acetone: *t *=* *−0.63, *df *=* *6, *p* = .552) and noxious (ethyl chloride: *t *=* *−0.62, *df *=* *6, *p* = .558) evaporative cooling, and heat stimulation (2‐way RM ANOVA, main effect: *F*_1,6_ = 0.052, *p *=* *.828; Figure 4d). However, in a neuropathic state DNIC were restored by inhibition of the ILC (23.2% decrease, Cohen's *d *=* *−0.897; 2‐way RM ANOVA, interaction: *F*_1,6_ = 25.60, *p* = .0023) (Figure 4e, f).

## 4 DISCUSSION

These data suggest that DRt involvement in DNIC requires an endogenous opioidergic mechanism, and secondly, the ILC is unlikely to be directly involved in mediating DNIC but can modulate its activation in differing pain states. These observations underscore the translational value of DNIC as an endpoint in rodent studies. Given that imaging studies cannot differentiate between excitatory and inhibitory neuronal activity, and the potential confound of expectations or attentional shifts in interpreting data, the current approach allows direct study of supra‐spinal influences on spinal sensory transmission at noxious intensities above withdrawal threshold.

Compared to brainstem nuclei, less is known about the role of cortical circuitry in descending modulation of pain. The mPFC is critical for executive functions and decision making, and cognitive impairment is commonplace in patients with chronic pain (Moriarty, McGuire, & Finn, 2011). Corticolimbic signalling assigns an emotional valence to sensory inputs (Corder et al., 2019; Thompson & Neugebauer, 2018), but mPFC projections to brainstem structures can also mediate top‐down regulation of sensory transmission (Cheriyan & Sheets, 2018; David‐Pereira et al., 2017; Jodo, Chiang, & Aston‐Jones, 1998). When a sensory signal is received, its salience must be determined and once the immediate threat is evaluated appropriate goal‐directed behaviours can be initiated. Following on from this, aversive learning guides future responses, and the prelimbic and infralimbic cortices in rodents mediate top‐down control of emotion‐driven behaviours such as fear conditioning and extinction (Giustino & Maren, 2015).

As revealed by silencing of the ILC, the current study supports that when two distant noxious stimuli are detected anti‐nociception is favoured; in chronic pain, where an ongoing aversive state exists, a shift may occur towards pro‐nociception. This may differ from the situation where a single noxious stimulus is given as no role of the ILC was observed on unconditioned responses. Prefrontal pyramidal neuronal excitability is suppressed in chronic inflammatory states, and much of this depressed activity derives from feedforward inhibition from GABAergic interneurones targeted by glutamatergic basolateral amygdala projections (Ji & Neugebauer, 2014; Ji et al., 2010). In addition, following nerve injury plasticity in cholinergic modulation can promote functional deactivation (Radzicki, Pollema‐Mays, Sanz‐Clemente, & Martina, 2017), and increased noradrenergic modulation drives aversive and anxiogenic behaviours (Hirschberg, Li, Randall, Kremer, & Pickering, 2017). Notably, both sensory and affective dimensions of pain can be ameliorated by augmenting this cortical activity as silencing GABAergic interneurones (Zhang et al., 2015), or optogenetic activation of pyramidal neurones (Lee et al., 2015), produces conditioned place preference in neuropathic animals in addition to reversing mechanical and thermal hypersensitivity.

In the absence of nerve injury, local lidocaine block of the ILC decreases heat‐evoked withdrawal latencies revealing tonic anti‐nociceptive function (David‐Pereira et al., 2016). We did not observe similar effects on the heat‐evoked neuronal endpoints in this study which could be attributed to the impact of anaesthesia on cortical–subcortical signalling. However, the abolition of DNIC was observed under these experimental conditions and is consistent with the ability of the ILC to engage descending inhibitory networks in a normal state. In the neuropathic rats, the most likely explanation is that inhibitory signalling from the ILC increases, and silencing this activity restores DNIC. Stimulation of cortical regions such as the ILC and anterior cingulate can exert pro‐nociceptive effects via the DRt revealing bidirectional control of nociceptive transmission (David‐Pereira et al., 2017; Zhang, Zhang, & Zhao, 2005). However, it would appear that the majority of GABAergic cortical projections to the DRt originate from the somatosensory, insula and motor cortices, and GABA release within the DRt is facilitatory by disinhibiting descending neurones (Martins et al., 2015a). It is also possible that intra‐ILC lidocaine disinhibits a wider descending pain modulatory network resulting in the restoration of DNIC, which may also involve the mid/anterior cingulate and the amygdala converging on final brainstem relays (Sprenger et al., 2011).

The DRt acts as an integrative relay for ascending sensory information projecting to multiple cortical regions but also receives extensive projections from the cortex, amygdala, locus coeruleus, rostral ventromedial medulla and periaqueductal grey (Almeida, Cobos, Tavares, & Lima, 2002; Bernard, Villanueva, Carroué, & Le Bars, 1990; Leite‐Almeida, Valle‐Fernandes, & Almeida, 2006). Anatomical, electrophysiological and behavioural evidence all support a descending facilitatory action of the DRt. Reciprocal connections between the DRt and spinal cord provide a neuroanatomical basis for nociceptive amplification (Almeida, Tavares, Lima, & Coimbra, 1993), and this brainstem–spinal cord circuit via the parabrachial nucleus controls nocifensive behaviours in response to noxious stimuli (Barik, Thompson, Seltzer, Ghitani, & Chesler, 2018). Both unilateral lesioning and bilateral lesioning of the DRt lead to an increase in the tail flick latency (Almeida, Tjolsen, Lima, Coimbra, & Hole, 1996) and an attenuation of formalin‐evoked nocifensive behaviours (Almeida, Storkson, Lima, Hole, & Tjolsen, 1999). Conversely, stimulating the DRt decreases the tail flick latency (Almeida et al., 1996) and increases the excitability of spinal wide dynamic range neurones (Dugast, Almeida, & Lima, 2003). Opioidergic interneurones within the DRt are positioned to provide feedback inhibition within the reticulospinal facilitatory loop, and these also express GABAB receptors (Martins et al., 2015a). We did not observe tonic opioidergic activity within the DRt in sham and SNL rats in response to acute noxious stimuli, however virally induced increases in endogenous enkephalin levels within the DRt produces hypoalgesia (Pinto et al., 2008), and this circuitry appears to be activated during DNIC as demonstrated by reversal with naloxone. The complexity of opioid systems in the circuits that regulate DNIC is supported by the finding that it is also attenuated by systemic morphine (Le Bars, Chitour, Kraus, Clot, et al., 1981), suggestive of concomitant inhibitory/disinhibitory actions at multiple sites.

Neurones within the DRt receive convergent Aδ‐ and C‐fibre input and exhibit whole body receptive fields (Villanueva, Bouhassira, Bing, & Le Bars, 1988), and a heterotopic noxious stimulus has a negative influence on neuronal activity (Villanueva, Bing, & Le Bars, 1994). Human studies have not always conclusively supported an endogenous opioidergic mechanism of CPM (Sprenger et al., 2011; Willer, Le Bars, & De Broucker, 1990), but DNIC in rats are partially reduced by systemic naloxone (Le Bars, Chitour, Kraus, Dickenson, et al., 1981), an effect that appears dependent on actions in the DRt, but independent of the rostral ventromedial medulla (de Resende, Silva, Sato, Arendt‐Nielsen, & Sluka, 2011). Given that individual DRt neurones can project to multiple targets, it is unclear how reduced neuronal activity within the DRt is permissive for DNIC. Cortical networks converging upon the DRt and the locus coeruleus could result in a reduction in facilitatory outflow from the former permitting inhibitory actions from the latter to predominate. However, direct interaction between these regions also occurs. Noradrenaline has an excitatory influence in the DRt via α_1_ adrenoceptors in neuropathic rats (Martins et al., 2015b). An alternate hypothesis could be a tonic inhibition from the DRt presiding over the locus coeruleus preventing DNIC, and thus, disinhibition would be permissive for DNIC to be fully activated. In neuropathy, a disrupted balance of activity in descending monoaminergic systems will also impact the expression of DNIC. Descending noradrenergic pathways remain intact after nerve injury but are hypoactive (Hirschberg et al., 2017; Hughes, Hickey, Hulse, Lumb, & Pickering, 2013; Patel, Qu, Xie, Porreca, & Dickenson, 2018), and DNIC are restored following spinal delivery of a noradrenaline reuptake inhibitor (Bannister et al., 2015). Chronic pain states can also be associated with increased descending facilitation, largely mediated via spinal 5‐HT_2A_ and 5‐HT_3_ receptors (Patel & Dickenson, 2018; Suzuki, Rahman, Hunt, & Dickenson, 2004), and enhanced excitatory drive can mask inhibitory signalling (Bannister et al., 2015; Nation et al., 2018; Okada‐Ogawa, Porreca, & Meng, 2009; Phelps, Navratilova, Dickenson, Porreca, & Bannister, 2019).

In summary, these data support the concordance of the mechanisms of CPM in humans and DNIC in rodents. These findings could form the basis of further explorations into cortical mechanisms of top‐down descending control of pain to identify pathophysiological mechanisms.

## CONFLICT OF INTEREST

The authors have no conflicts of interest to declare.

## AUTHOR CONTRIBUTIONS

RP and AHD conceived and designed the study; AHD provided experimental equipment and resources; RP collected and analysed data; RP and AHD interpreted results of experiments; RP prepared figures; RP drafted the manuscript; and RP and AHD edited and revised the manuscript. Both authors read and approved the final manuscript.

## Data Availability

Data sets are available online (https://doi.org/10.6084/m9.figshare.9785816).
